# Data on the characterization and anticancer action of iron(II) polypyridyl complexes

**DOI:** 10.1016/j.dib.2016.05.030

**Published:** 2016-06-22

**Authors:** Jingjing Chen, Zuandi Luo, Zhennan Zhao, Lina Xie, Wenjie Zheng, Tianfeng Chen

**Affiliations:** Department of Chemistry, Jinan University, Guangzhou 510632, China

## Abstract

This data article contains complementary figures and results related to the research article entitled, “Cellular localization of iron(II) polypyridyl complexes determines their anticancer action mechanisms” [1] (Chen et al., 2015).

The characterization of Fe(II) complexes by ESI-MS, ^1^H NMR, ^13^C NMR spectroscopy, FT-IR spectra, UV–vis spectra was provided. Also,the data for the stability of Fe(II) complexes **1–5** in DMSO/Milli-Q water/ culture medium (without serum or phenol red) at 37 °C at different periods of time by UV–vis spectra and ^1^H NMR was showed. At the same time, the anticancer efficacy, cellular distribution and ROS generation in MCF-7 cells of complexes are reported. In addition, we also show the cellular localization of complex **4**, the relative fluorescence intensity of complex **1** and complex **3** pretreated with anti-TfR (2 μg/mL) in MCF-7 cells using flow cytometry. The compilation of this data provides an invaluable resource for the wider research community and the interpretation of these data could be found in the research article noted above.

**Specifications Table**

**Table:** 

Subject area	*Chemistry*
More specific subject area	*Metal complexes, cellular localization, anticancer mechanism*
Type of data	*Table, image, graph, figure*
How data was acquired	*NMR, mass spectroscopy,UV–vis, FT-IR, MTT assay, fluorescence microscope, flow cytometry, DHE assay.*
Data format	*Raw, analyzed*
Experimental factors	*The stability of the Fe(II) complexes was carried out at 37* *°C; MCF-7 cells were exposed to the different concentrations of the Fe(II) complexes for different periods of time and analyzed by MTT assay; cells were treated with complex **4** and examined under fluorescence microscope; MCF-7 cells were treated with Fe(II) complexes and analysis its distribution; MCF-7 cells pretreated with anti-TfR, and then Fe(II) complexes were added and sequentially incubated in CO*_*2*_*incubator, the results were analyzed by flow cytometry; MCF-7 cells harvested by centrifugation and resuspended in PBS were incubated with DHE, then the cells were incubated with different concentrations of complexes for different periods of time.*
Experimental features	*The characterization of the complexes was analyzed by ESI-MS,*^*1*^*H NMR,*^*13*^*C NMR spectroscopy, FT-IR spectra, UV–vis spectra; the stability of the Fe(II) complexes was carried out by*^*1*^*H NMR and UV–vis spectra; the anticancer efficacy was carried out by MTT assay; the cellular localization of complex **4** in MCF-7, A375 and HeLa was examined under fluorescence microscope; the cellular distribution of Fe complexes was analysis by fluorescence intensity; the relative fluorescence intensity of complex **1** and complex **3** pretreated with anti-TfR (2* μ*g/mL) in MCF-7 cells were analyzed by flow cytometry; the ROS generation in MCF-7 cells of complexes was measured by DHE assay.*
Data source location	*Guangzhou, China*
Data accessibility	*All data provided within this article*

**Value of the data**

• Data on ESI-MS, ^1^H NMR, ^13^C NMR spectroscopy, FT-IR spectra and UV–vis spectra were provided for the characterization of Fe(II) complexes, which provides an invaluable resource for the wider research community.
• Data on UV–vis spectra and ^1^H NMR in DMSO, aqueous media and culture medium during incubation at 37 °C within 72 h, which show the complexes are stable once internalized by the cells, opening up doors for new collaborations.
• Data on anticancer efficacy, cellular ROS levels, cellular distribution and cellular localization of complexes was provided, which was available for other researchers using this data as a benchmark.

## 1 Data

In this article we share the synthesis and characterization of Fe(II) complexes **1–5** that exhibit potent anticancer activities [1]. The stability, cytotoxicity, intracellular ROS generation levels, cellular distribution, cellular localization and relative fluorescence intensity of complexes were shown See Table 1 and Fig. 1, Fig. 2, Fig. 3, Fig. 4, Fig. 5, Fig. 6, Fig. 7, Fig. 8, Fig. 9, Fig. 10, Fig. 11, Fig. 12, Fig. 13, Fig. 14, Fig. 15, Fig. 16, Fig. 17, Fig. 18, Fig. 19, Fig. 20, Fig. 21, Fig. 22, Fig. 23, Fig. 24.

## 2 Experimental design, materials and methods

### 2.1 Methods

#### 2.1.1 The characterization of the Fe(II) complexes 1 and 2

*Synthesis of Fe(bpy)_3_(ClO_4_)_2_ (1) and Fe(phen)_3_(ClO_4_)_2_ (2)***.** The Fe(II) complexes 1–2 were synthesized according to published procedures [2]. **Fe(bpy)**_**3**_**(ClO**_**4**_**)**_**2**_ **(1)** Yield 85%. ESI-MS: *m*/*z* 261.9 [M-2(ClO_4_^−^)]^2+^. Elemental analysis calc (%) for C_30_H_24_Cl_2_N_6_O_8_Fe: C, 49.82; H, 3.34; N, 11.62; found (%): C, 49.76; H, 3.28; N, 15.50. UV–vis (*λ* (nm), *ε*/10^4^ (M^−1^ cm^−1^): 295 (6.3), 518 (0.66). IR (KBr): ***ν*** 3090 (N-H), ***ν*** 1610, 1440 (C=C _arom_) cm^−1^. ^1^H NMR (DMSO-d_6_, δ ppm): 8.87 (d, 6H), 8.22 (t, 6H), 7.53 (t, 6H), 7.42 (d, 6H). ^13^C NMR (DMSO-d_6_, δ ppm): 159.21 (s, 6C), 154.12 (s, 6C), 139.07 (s, 6C), 128.03 (s, 6C), 124.57(s, 6C).

**Fe(phen)**_**3**_**(ClO**_**4**_**)**_**2**_ **(2)** Yield 90%. ESI-MS: *m*/*z* 298.07 [M-2(ClO_4_^−^)]^2+^. Elemental analysis calc (%) for C_36_H_24_Cl_2_N_6_O_8_Fe: C, 54.36; H, 3.04; N, 10.57; found (%): C, 54.30; H, 2.97; N, 10.51. UV–vis (λ (nm), ε/10^4^ (M^−1^ cm^−1^): 264 (9.47), 511 (0.98). IR (KBr): ***ν*** 3060 (N–H), ***ν*** 1630, 1425 (C=C_arom_) cm^−1^. ^1^H NMR (DMSO-d_6_, δ ppm): 8.81 (d, 6H), 8.41 (s, 6H), 7.75 (t, 6H), 7.72 (d, 6H). ^13^C NMR (DMSO-d_6_, δ ppm): 156.53 (s, 6C), 149.87 (s, 6C), 137.97 (s, 6C), 130.32 (s, 6C), 128.83(s, 6C), 126.91(s, 6C).

#### 2.1.2 Stability of Fe(II) complexes

The stability of the Fe(II) complexes in DMSO, aqueous media, culture medium were examined by UV–vis spectrometry using a Cary 5000 UV-2450 spectropho-tometer. Spectra was collected from samples dissolved in DMSO/Milli-Q water/ culture medium (without serum or phenol red). Each spectrum (230–600 nm) was recorded after incubation of the sample in DMSO/Milli-Q water/ culture medium (without serum or phenol red) at 37 °C at different periods of time.

The stability of the Fe(II) complexes in aqueous media was also examined by.

^1^H NMR (Bruker, 300 MHz). Nuclear magnetic resonance spectroscopy was collected from samples dissolved in deuteroxide. Each spectrum was recorded after incubation of the sample in deuteroxide at different periods of time.

#### 2.1.3 Examination the distribution of Fe complexes in MCF-7 cells

MCF-7 cells were treated with 32 μM of Fe(II) complexes for 24 h respectively, and separated the nucleus by Nuclei PURE Prep (NUC201-1KT). The collected fractions were then subjected to fluorescence determination.

#### 2.1.4 Measurement of ROS generation

The intracellular ROS generation levels in MCF-7 cells by complexes **1–5** were measured by DHE assay as reported [3]. ROS generation was measured by the fluorescence intensity on a Tecan SAFIRE fluorescence reader, the excitation and emission wavelengths were 300 and 610 nm. Relative DHE fluorescence intensity of treated cells was expressed as percentage of control (as 100%).

## Figures and Tables

**Figure:**
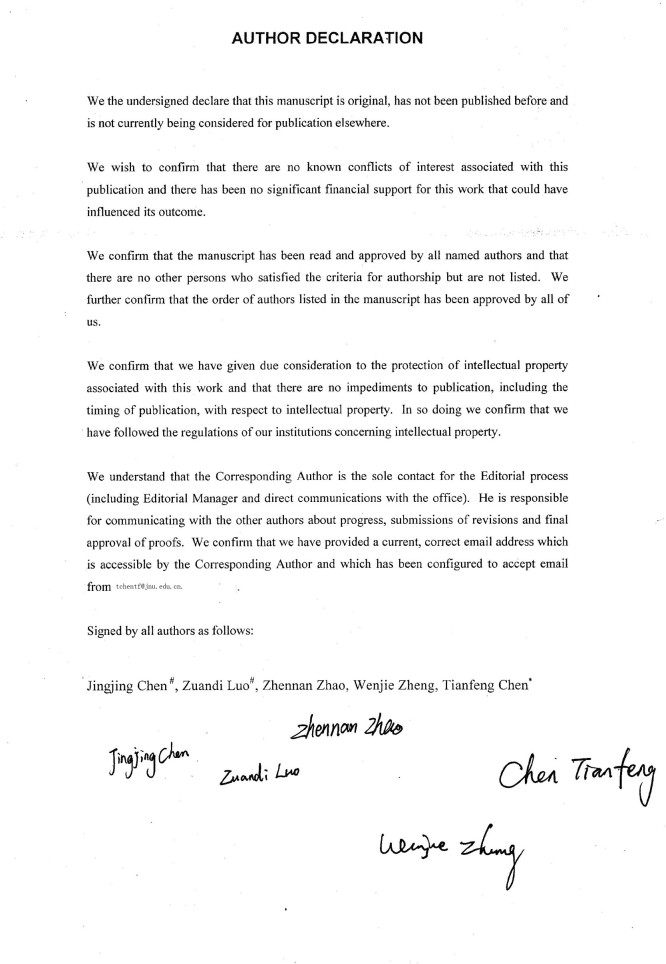
Supplementary material

**Fig. 1:**
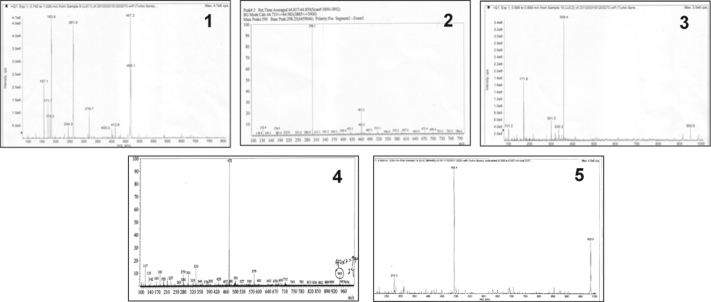
The ESI-MS of complexes **1–5**.

**Fig. 2:**
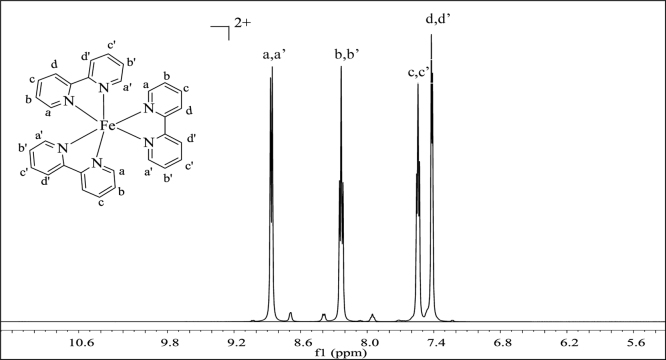
^1^H NMR spectrum (500 MHz) of complex **1** in dimethylsulfoxide-d^6^.

**Fig. 3:**
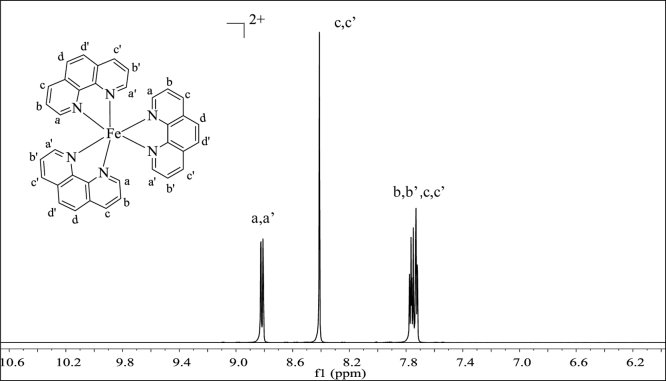
^1^H NMR spectrum (500 MHz) of complex **2** in dimethylsulfoxide-d^6^.

**Fig. 4:**
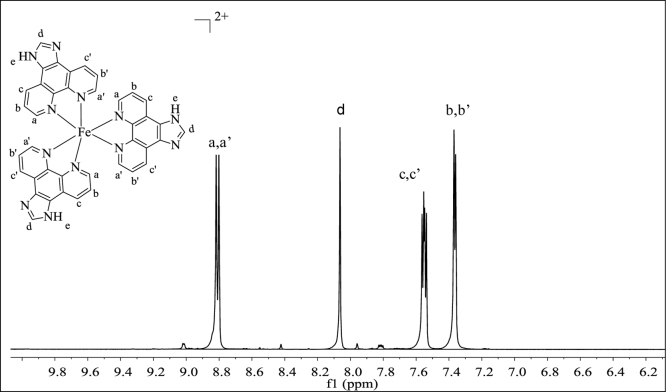
^1^H NMR spectrum (500 MHz) of complex **3** in dimethylsulfoxide-d^6^.

**Fig. 5:**
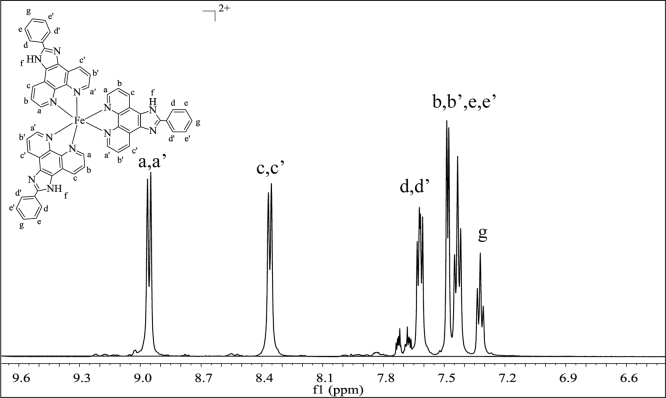
^1^H NMR spectrum (500 MHz) of complex **4** in dimethylsulfoxide-d^6^.

**Fig. 6:**
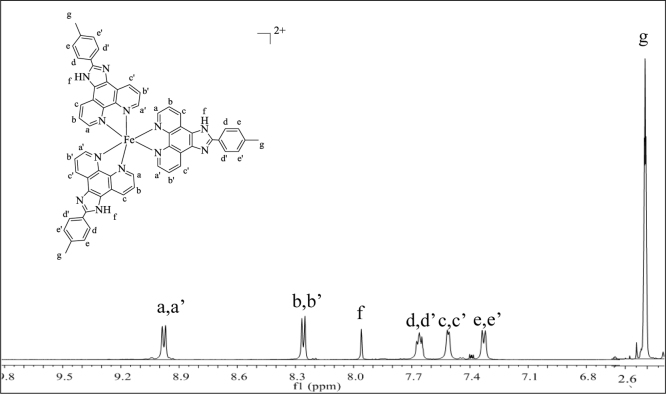
^1^H NMR spectrum (500 MHz) of complex **5** in dimethylsulfoxide-d^6^.

**Fig. 7:**
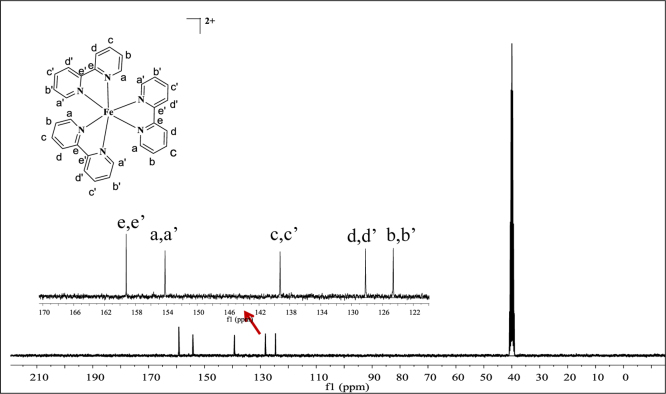
^13^C NMR spectrum (300 MHz) of complex **1** in dimethylsulfoxide-d^6^.

**Fig. 8:**
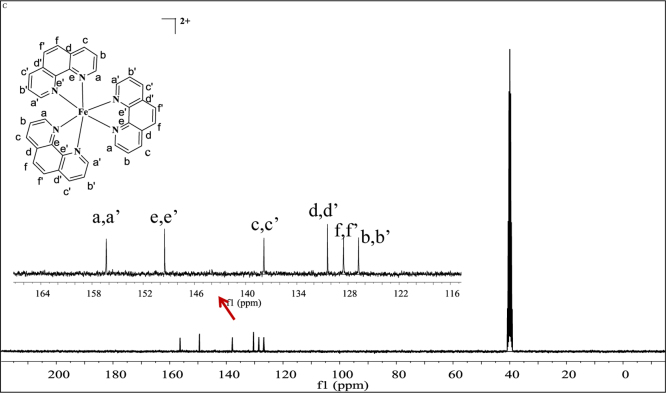
^13^C NMR spectrum (300 MHz) of complex **2** in dimethylsulfoxide-d^6^.

**Fig. 9:**
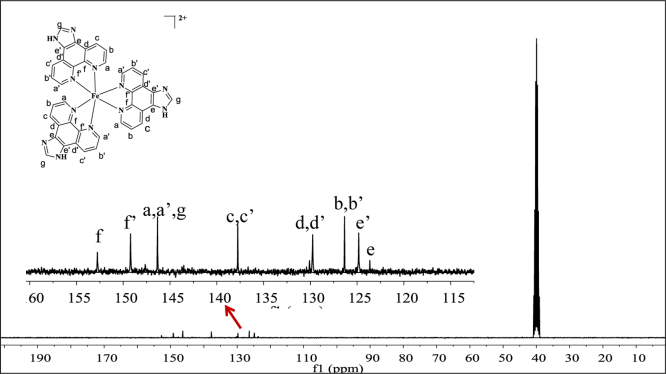
^13^C NMR spectrum (300 MHz) of complex **3** dimethylsulfoxide-d^6^.

**Fig. 10:**
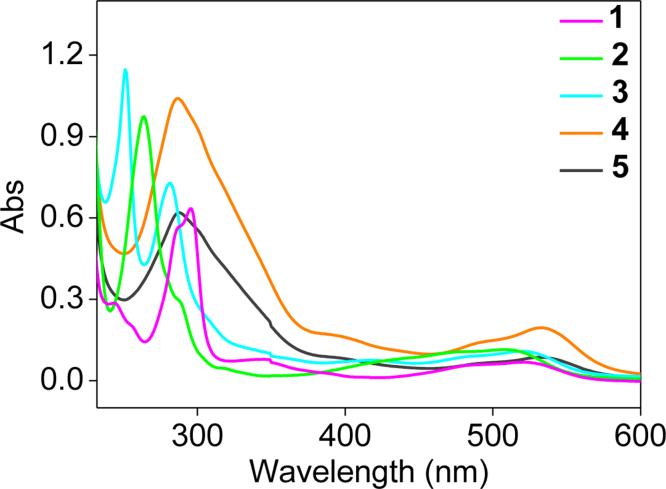
UV–visible spectrum of the Fe(II) complexes 1–5 in 5 mM Tris–HCl buffer (pH=7.2).

**Fig. 11:**
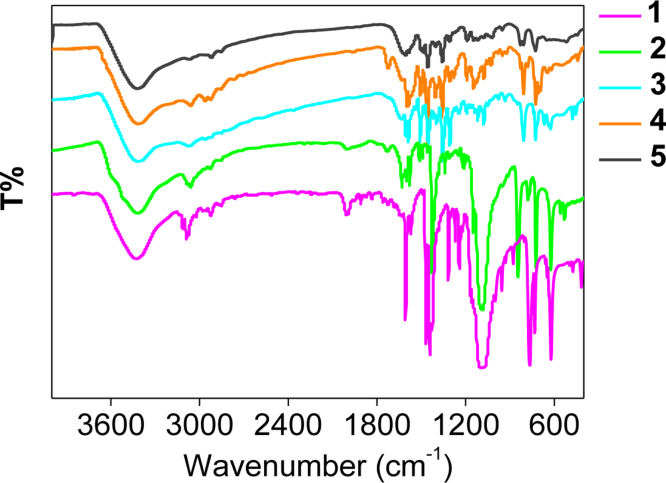
FT-IR spectrum of the Fe(II) complexes 1–5 in KBr phase.

**Fig. 12:**
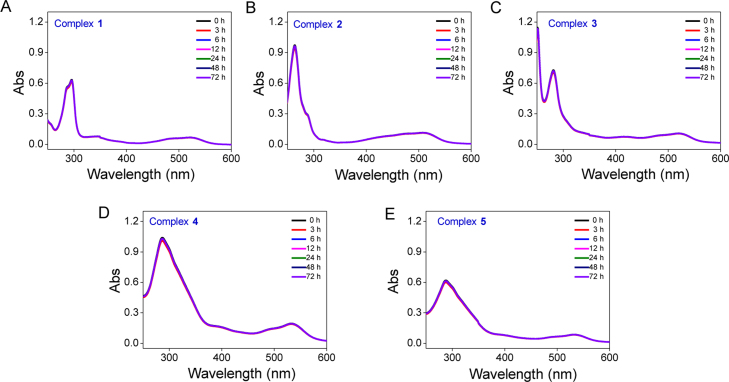
The stability of Fe(II) complexes **1–5** in DMSO during incubation at 37 °C within 72 h. **(**A) **1**, (B) **2**, (C) **3**, (D) **4**, (E) **5**.

**Fig. 13:**
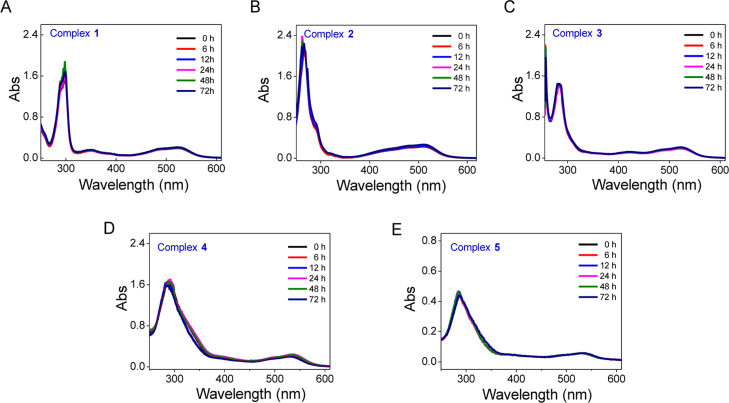
The stability of Fe(II) complexes **1–5** in H_2_O during incubation at 37 °C within 72 h. **(**A) **1**, (B) **2**, (C) **3**, (D) **4**, (E) **5**.

**Fig. 14:**
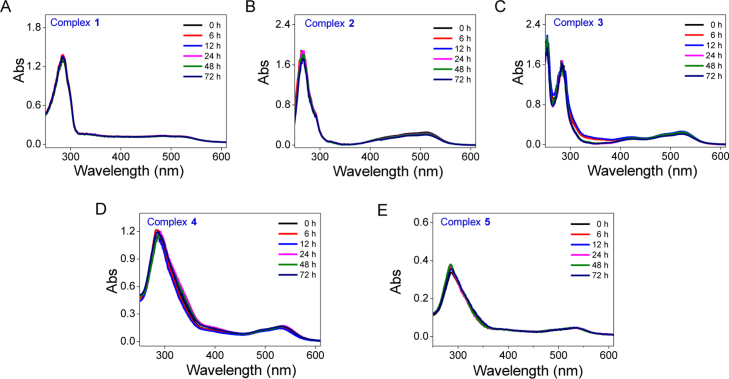
The stability of Fe(II) complexes **1–5** in DMEM during incubation at 37 °C within 72 h. **(**A) **1**, (B) **2**, (C) **3**, (D) **4**, (E) **5**.

**Fig. 15:**
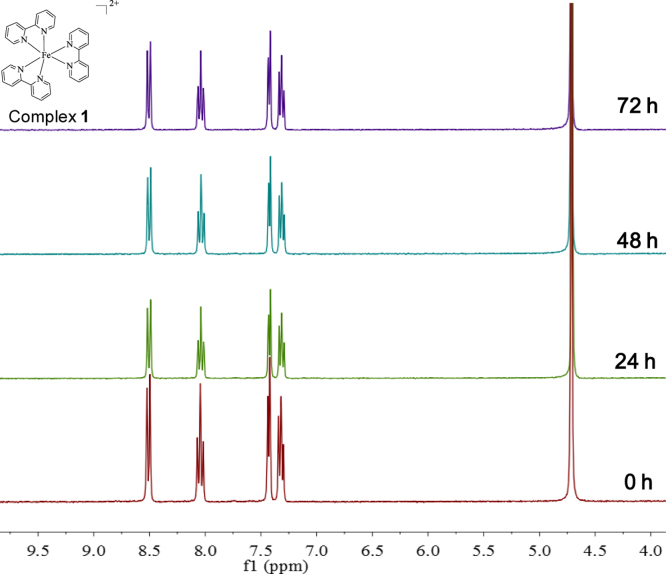
^1^H NMR spectrum (300 MHz) of complex **1** in deuterium oxide within 72 h.

**Fig. 16:**
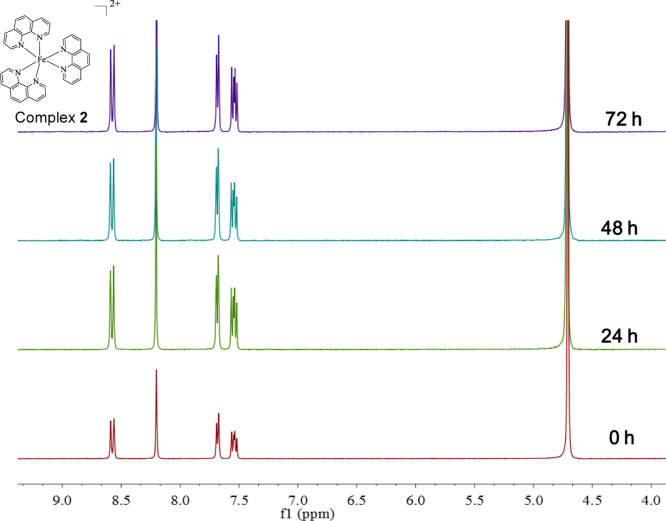
^1^H NMR spectrum (300 MHz) of complex **2** in deuterium oxide within 72 h.

**Fig. 17:**
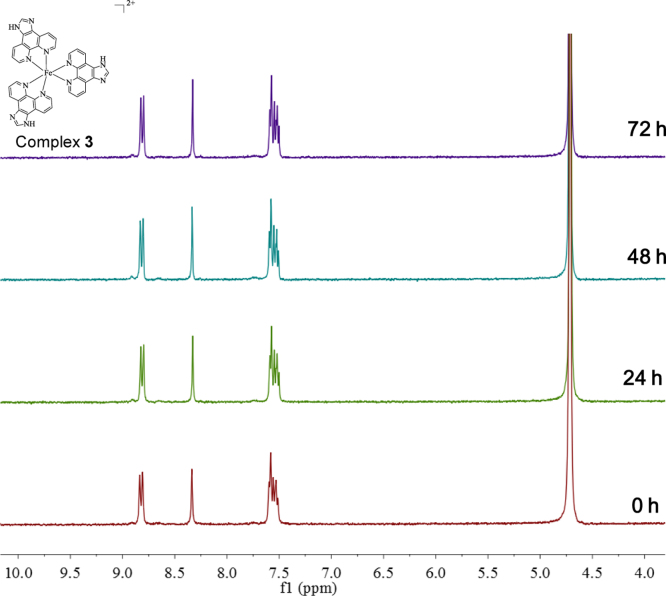
^1^H NMR spectrum (300 MHz) of complex **3** in deuterium oxide within 72 h.

**Fig. 18:**
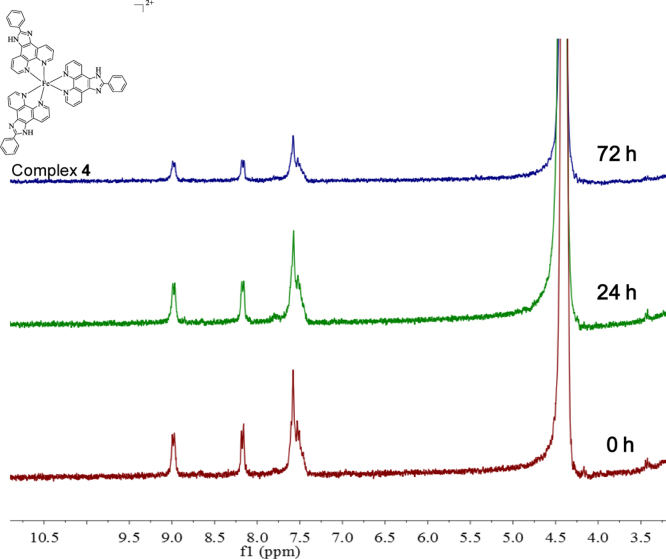
^1^H NMR spectrum (300 MHz) of complex **4** in deuterium oxide within 72 h.

**Fig. 19:**
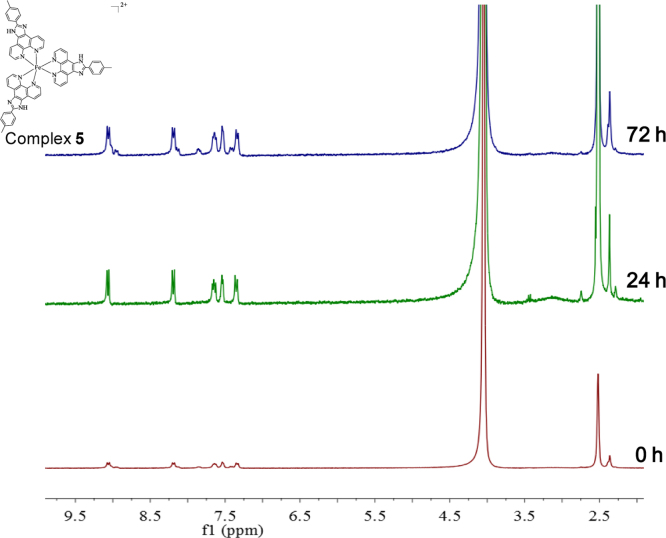
^1^H NMR spectrum (300 MHz) of complex **5** in deuterium oxide within 72 h.

**Fig. 20:**
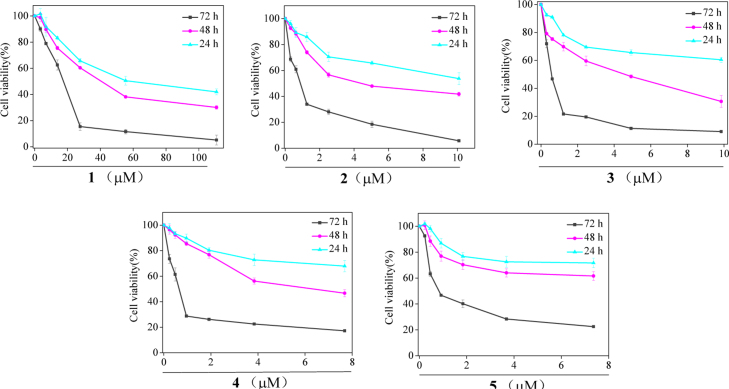
The time- and dose-dependent cytotoxicity of Fe(II) complexes **1–5.** MCF-7 cells were exposed to the different concentrations of the Fe(II) complexes **1–5** for 24 h, 36 h, 72 h.

**Fig. 21:**
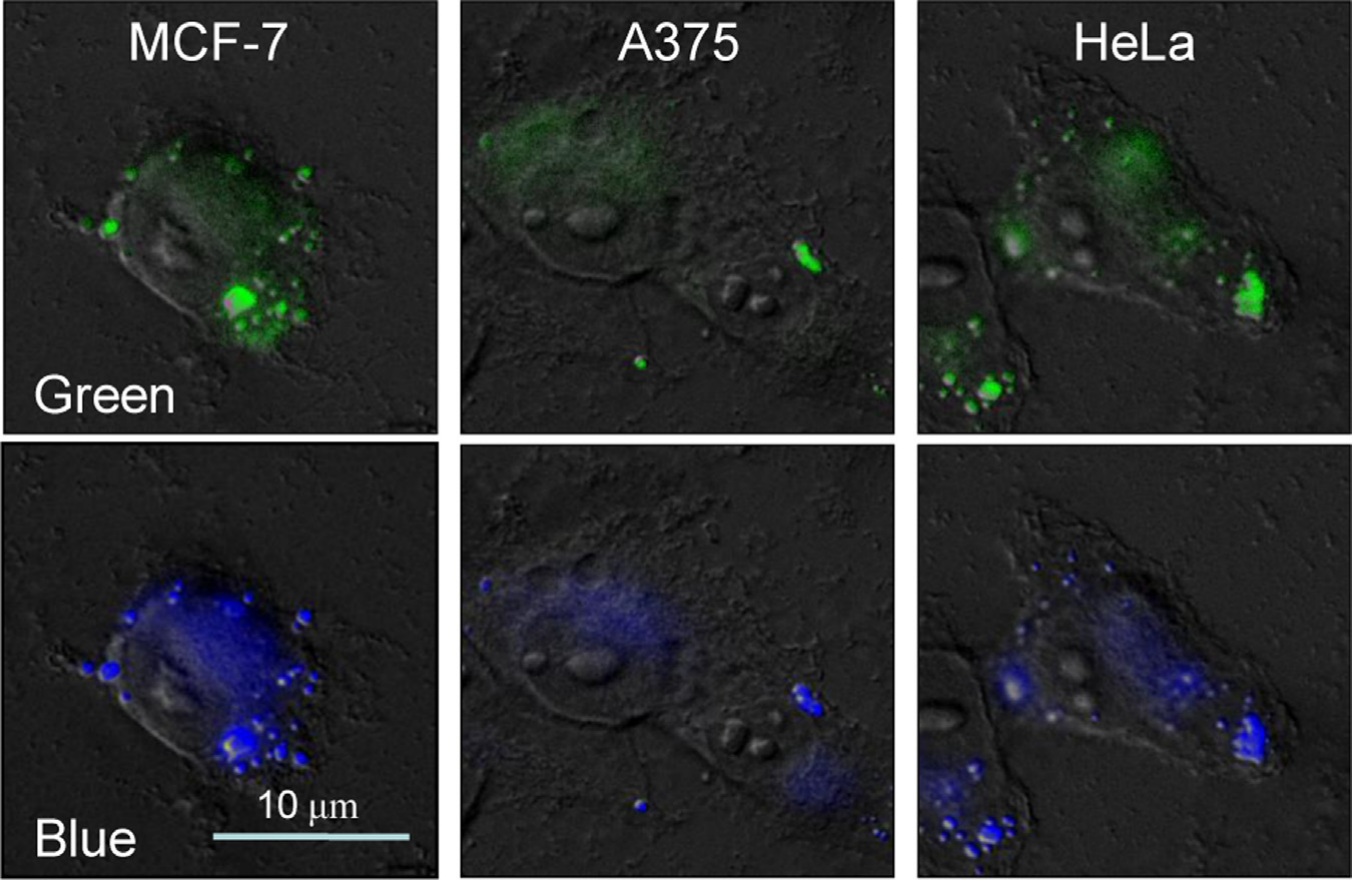
The cellular localization of complex **4** in MCF-7, A375 and HeLa cells respectively. Cells were treated with 32 μM of complex **4** for 24 h and examined under fluorescence microscope.

**Fig. 22:**
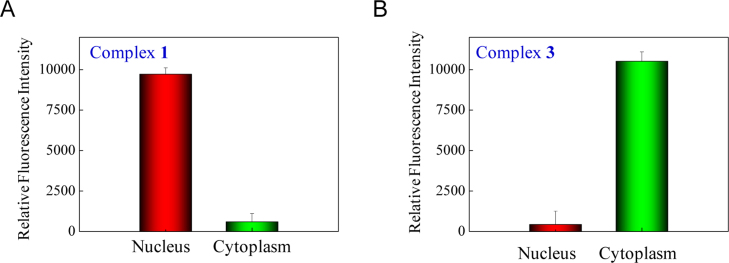
The distribution of Fe complexes (A) **1**, (B) **3** in MCF-7 cells. MCF-7 cells were treated with 32 μM of Fe(II) complexes for 24 h respectively.

**Fig. 23:**
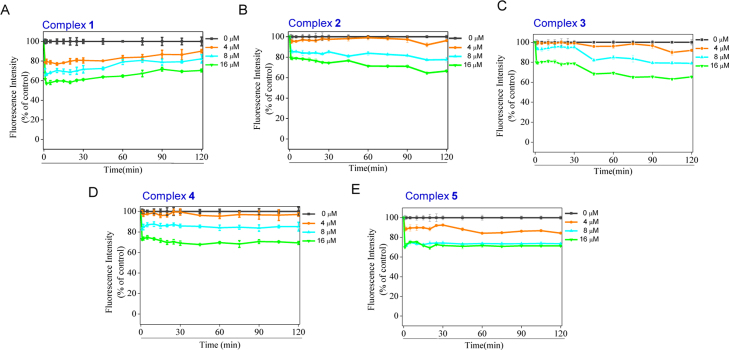
The intracellular ROS generation levels in MCF-7 cells by complexes **1–5** using DHE assay.(A) complex **1**, (B) complex **2**, (C) complex **3**, (D) complex **4** and (E) complex **5**.

**Fig. 24:**
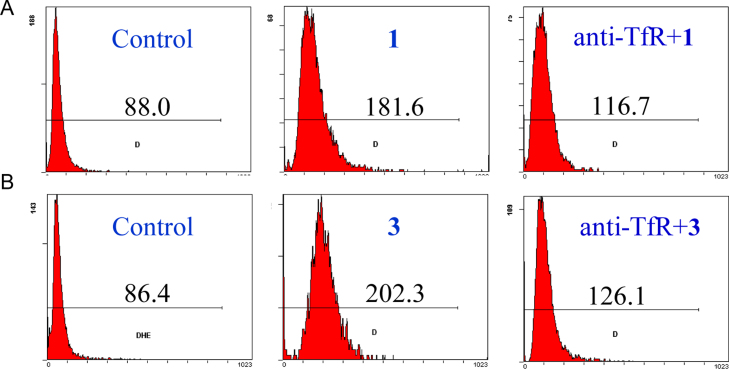
Relative fluorescence intensity of (A) complex **1 (**32 μM, 12 h) and (B) complex **3 (**32 μM, 12 h) pretreated with anti-TfR (2 μg/mL, 2 h) in MCF-7 cells using flow cytometry.

**Table 1:** The ESI-MS analysis of complexes **1–5**.

Complexes	Theoretical value (*m*/*z*)	Measured value (*m*/*z*)	Belonging to (*m*/*z*)
**1**	262.07	261.9	${{[M-2(\text{ClO}}_{4}^{-})]}^{2+}$
**2**	298.07	298.07	${{[M-2(\text{ClO}}_{4}^{-})]}^{2+}$
**3**	358.08	358.4	${{[M-(\text{SO}}_{4}^{2-})]}^{2+}$
**4**	472.12	472.0	${{[M-(\text{SO}}_{4}^{2-})]}^{2+}$
**5**	493.15	493.4	${{[M-(\text{SO}}_{4}^{2-})]}^{2+}$
